# Supplementary material for: β-lactam Resistance in Pseudomonas aeruginosa: Current Status, Future Prospects
Source: Pathogens. 2021 Dec 18;10(12):1638. doi: 10.3390/pathogens10121638 (PMC8706265; doi:10.3390/pathogens10121638)
Supplement: Supplementary file 1 [file pathogens-10-01638-s001.zip › pathogens-1468556-supplementary.pdf]

Supplementary Material

Table S1. *P. aeruginosa* PBP3 variants within the transpeptidase domain[a.]

Variants	N242S	A244S/T	A246V	V254M	G257R	T267A	R284H	P298S	Y367C	S368L	Q372P	H394R	A419G	N427K/S	L434V	A454D/V	M460V	L461V	V464A	V465A/G	E466L	Q468L	V471L/G	Q475R	A482V	K490M	Y503H	R504C/H/L	S505P	F507L/V	A513T	V523L/A/M	I524T/S	P527S/T	G531D/E	F533L	V537L	S538L/P	A539T	V541M	S543G	Study
**Clinical Isolates**																																										
										red		blue	red	red	blue	red		blue						blue				red		red				red				red				1
																												red								red						2
	blue	red				blue			red																red			red			blue		red	red	blue						blue	3
											red																	red						red								4
																												red														5
		red																		red														red								6
																																		red	blue							7
							red	red	red			blue															red	red				red	red		blue	red		red				8
					red																							red														9
																red	red						blue													red						10
																							blue					red														11
																												red														12
									red			blue		red										blue				blue				red (hatched)				red						13
		red		blue		blue						blue							blue		red					red		red	red	red			red	red		red	red			red		14
																												red														15
**Experimentally evolved**																																										
	blue	red	red																			red												red								3
																				red								blue								red			red			16
																							blue														red					17

[a]Sequence variants in the transpeptidase domain of PBP3 in clinical isolates and experimentally evolved -lactam-resistant mutants are listed. Variants predicted using the bioinformatics tool Provean [1, 17, 18] to affect protein function are shown in red, and those predicted to not significantly affect protein function are in blue. The table is derived from a figure from[1].

Table S2. *P. aeruginosa* PBP3 variants outside the transpeptidase domain[a]

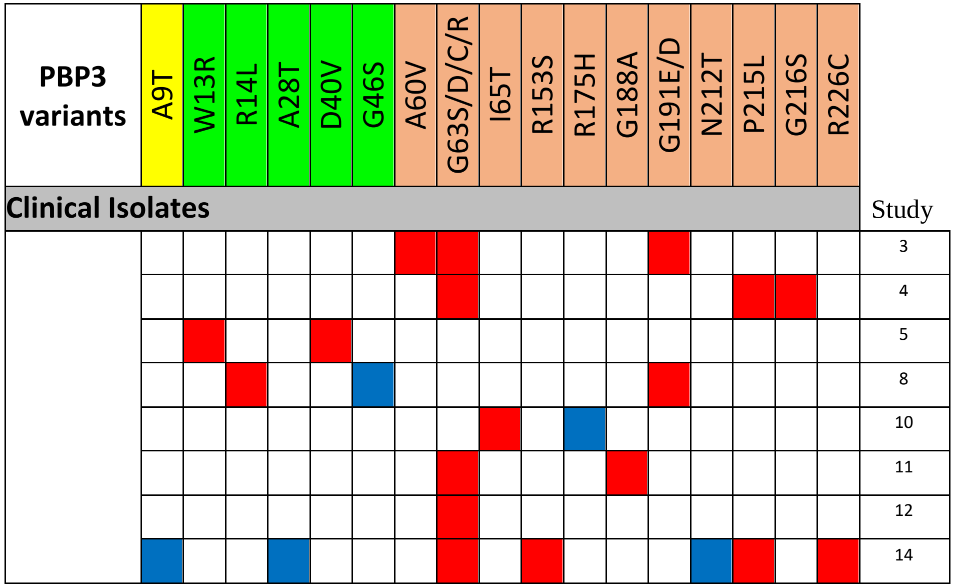

[a] Sequence variants in the cytoplasmic (yellow), membrane spanning alpha-helix (green) and domain likely involved in protein-protein interactions (Brown) of PBP3 in clinical isolates and experimentally evolved β-lactam-resistant mutants are listed. Variants predicted using the bioinformatics tool Provean [1, 17, 18] to affect protein function are shown in red, and those predicted to not significantly affect protein function in blue. Figure is derived from a figure from [1].

References

1. Clark, S. T.; Sinha, U.; Zhang, Y.; Wang, P. W.; Donaldson, S. L.; Coburn, B.; Waters, V. J.; Yau, Y. C. W.; Tullis, D. E.; Guttman, D. S.; Hwang, D. M., Penicillin-binding protein 3 is a common adaptive target among *Pseudomonas aeruginosa* isolates from adult cystic fibrosis patients treated with β-lactams. *International Journal of Antimicrobial Agents* **2019,** *53* (5), 620-628.
2. del Barrio-Tofiño, E.; López-Causapé, C.; Cabot, G.; Rivera, A.; Benito, N.; Segura, C.; Montero, M. M.; Sorlí, L.; Tubau, F.; Gómez-Zorrilla, S., Genomics and susceptibility profiles of extensively drug-resistant *Pseudomonas aeruginosa* isolates from Spain. *Antimicrobial Agents and Chemotherapy* **2017,** *61* (11), e01589-17.
3. Jorth, P.; McLean, K.; Ratjen, A.; Secor, P. R.; Bautista, G. E.; Ravishankar, S.; Rezayat, A.; Garudathri, J.; Harrison, J. J.; Harwood, R. A., Evolved aztreonam resistance is multifactorial and can produce hypervirulence in *Pseudomonas aeruginosa*. *mBio* **2017,** *8* (5), e00517-17.

4. López-Causapé, C.; Sommer, L. M.; Cabot, G.; Rubio, R.; Ocampo-Sosa, A. A.; Johansen, H. K.; Figuerola, J.; Cantón, R.; Kidd, T. J.; Molin, S., Evolution of the *Pseudomonas aeruginosa* mutational resistome in an international cystic fibrosis clone. *Scientific Reports* **2017,** *7* (1), 1-15.
5. Cabot, G.; Lopez-Causape, C.; Ocampo-Sosa, A. A.; Sommer, L. M.; Dominguez, M. A.; Zamorano, L.; Juan, C.; Tubau, F.; Rodriguez, C.; Moya, B.; Pena, C.; Martinez-Martinez, L.; Plesiat, P.; Oliver, A., Deciphering the resistome of the widespread *Pseudomonas aeruginosa* sequence type 175 international high-risk clone through whole-genome sequencing. *Antimicrobial Agents and Chemotherapy* **2016,** *60* (12), 7415-7423.
6. Diaz Caballero, J.; Clark, S. T.; Coburn, B.; Zhang, Y.; Wang, P. W.; Donaldson, S. L.; Tullis, D. E.; Yau, Y. C.; Waters, V. J.; Hwang, D. M.; Guttman, D. S., Selective sweeps and parallel pathoadaptation drive *Pseudomonas aeruginosa* evolution in the cystic fibrosis lung. *mBio* **2015,** *6* (5), e00981-15.
7. Marvig, R. L.; Sommer, L. M.; Molin, S.; Johansen, H. K., Convergent evolution and adaptation of *Pseudomonas aeruginosa* within patients with cystic fibrosis. *Nature Genetics* **2015,** *47* (1), 57-64.
8. Jorth, P.; Staudinger, B. J.; Wu, X.; Hisert, K. B.; Hayden, H.; Garudathri, J.; Harding, C. L.; Radey, M. C.; Rezayat, A.; Bautista, G.; Berrington, W. R.; Goddard, A. F.; Zheng, C.; Angermeyer, A.; Brittnacher, M. J.; Kitzman, J.; Shendure, J.; Fligner, C. L.; Mittler, J.; Aitken, M. L.; Manoil, C.; Bruce, J. E.; Yahr, T. L.; Singh, P. K., Regional isolation drives bacterial diversification within cystic fibrosis lungs. *Cell Host Microbe* **2015,** *18* (3), 307-319.
9. Markussen, T.; Marvig, R. L.; Gómez-Lozano, M.; Aanæs, K.; Burleigh, A. E.; Høiby, N.; Johansen, H. K.; Molin, S.; Jelsbak, L., Environmental heterogeneity drives within-host diversification and evolution of *Pseudomonas aeruginosa*. *mBio* **2014,** *5* (5), e01592-14.
10. Marvig, R. L.; Johansen, H. K.; Molin, S.; Jelsbak, L., Genome analysis of a transmissible lineage of *Pseudomonas aeruginosa* reveals pathoadaptive mutations and distinct evolutionary paths of hypermutators. *PLOS Genetics* **2013,** *9* (9), e1003741.
11. Yang, L.; Jelsbak, L.; Marvig, R. L.; Damkiaer, S.; Workman, C. T.; Rau, M. H.; Hansen, S. K.; Folkesson, A.; Johansen, H. K.; Ciofu, O.; Hoiby, N.; Sommer, M. O.; Molin, S., Evolutionary dynamics of bacteria in a human host environment. *Proceedings of the National Academy of Sciences of the United States of America* **2011,** *108* (18), 7481-7486.
12. Chotirmall, S. H.; Sherrard, L. J.; Tai, A. S.; Wee, B. A.; Ramsay, K. A.; Kidd, T. J.; Ben Zakour, N. L.; Whiley, D. M.; Beatson, S. A.; Bell, S. C., Within-host whole genome analysis of an antibiotic resistant *Pseudomonas aeruginosa* strain sub-type in cystic fibrosis. *PLOS ONE* **2017,** *12* (3).
13. Colque, C. A.; Albarracin Orio, A. G.; Feliziani, S.; Marvig, R. L.; Tobares, A. R.; Johansen, H. K.; Molin, S.; Smania, A. M., Hypermutator *Pseudomonas aeruginosa* exploits multiple genetic pathways to develop multidrug resistance during long-term infections in the airways of cystic fibrosis patients. *Antimicrobial Agents and Chemotherapy* **2020**.
14. McLean, K.; Lee, D.; Holmes, E. A.; Penewit, K.; Waalkes, A.; Ren, M.; Lee, S. A.; Gasper, J.; Manoil, C.; Salipante, S. J., Genomic analysis identifies novel *Pseudomonas aeruginosa* resistance genes under selection during inhaled aztreonam therapy in vivo. *Antimicrobial Agents and Chemotherapy* **2019,** *63* (9), e00866-19.
15. Khaledi, A.; Weimann, A.; Schniederjans, M.; Asgari, E.; Kuo, T.-H.; Oliver, A.; Cabot, G.; Kola, A.; Gastmeier, P.; Hogardt, M.; Jonas, D.; Mofrad, M. R.; Bremges, A.; McHardy, A. C.; Häussler, S., Predicting antimicrobial resistance in *Pseudomonas aeruginosa* with machine learning-enabled molecular diagnostics. *EMBO Molecular Medicine* **2020,** *12* (3), e10264.
16. Cabot, G.; Zamorano, L.; Moya, B.; Juan, C.; Navas, A.; Blazquez, J.; Oliver, A., Evolution of *Pseudomonas aeruginosa* antimicrobial resistance and fitness under low and high mutation rates. *Antimicrobial Agents and Chemotherapy* **2016,** *60* (3), 1767-1778.
17. Wardell, S. J. T.; Rehman, A.; Martin, L. W.; Winstanley, C.; Patrick, W. M.; Lamont, I. L., A large-scale whole-genome comparison shows that experimental evolution in response to antibiotics predicts changes in naturally evolved clinical *Pseudomonas aeruginosa*. *Antimicrobial Agents and Chemotherapy* **2019**, e01619-19.
18. Choi, Y.; Sims, G. E.; Murphy, S.; Miller, J. R.; Chan, A. P., Predicting the functional effect of amino acid substitutions and indels. **2012**.
